# Effect of standardized post-coercion review session on symptoms of PTSD: results from a randomized controlled trial

**DOI:** 10.1007/s00406-020-01215-x

**Published:** 2020-11-24

**Authors:** Alexandre Wullschleger, Angelika Vandamme, Juliane Mielau, Lara Renner, Felix Bermpohl, Andreas Heinz, Christiane Montag, Lieselotte Mahler

**Affiliations:** 1 Department of Psychiatry and Psychotherapy, Berlin Institute of Health, Charité Universitätsmedizin Berlin, Corporate Member of Freie Universität Berlin, Humboldt-Universität Zu Berlin, Campus Charité Mitte, Berlin, Germany; 2 Division of Adult Psychiatry, Department of Psychiatry, Geneva University Hospitals, 2, Chemin du Petit-Bel-Air, 1226 Thonex, Switzerland; 3 Steinbeis Transfer Institute Medical Psychology, Steinbeis-Hochschule Berlin, Berlin, Germany

**Keywords:** Post coercion review, Coercion, PTSD, Trauma, Psychosis

## Abstract

**Objective:**

Post-coercion review is increasingly regarded as a mean to reduce the negative consequences of coercive interventions, including the development of posttraumatic symptoms. However, the efficacy of this intervention in preventing posttraumatic symptoms or PTSD has not been sufficiently studied. The objective of this study is to examine the influence of a single, standardized post-coercion review session on the development or exacerbation of PTSD symptoms in patients with psychotic disorders.

**Methods:**

In a multi-center, two-armed, randomized controlled trial, patients who experienced coercive interventions during current hospitalization were either randomized to standard treatment or an intervention group receiving a guideline-based, standardized reflecting review session. Factorial MANCOVA and subsequent ANCOVAs investigated the effects of the post-coercion reflecting review session on post-traumatic symptoms as measured by the subscales of the Impact of Events Scale-Revised (IES-R). Similarly, the effect of the intervention on the intensity of the peritraumatic reactions measured by the Peritraumatic Distress Inventory (PDI) was analyzed by conducting a factorial ANCOVA.

**Results:**

*N* = 82 patients were included in an intention-to-treat analysis. MANCOVA and post hoc ANCOVAs revealed a significant main effect of the intervention for the IES-R subscales intrusion and hyperarousal, when controlling for levels of peritraumatic distress, whereby intervention group participants presented lower respective mean scores. There was no significant difference regarding the intensity of the peritraumatic reaction.

**Conclusion:**

Standardized post-coercion review contributes to a reduction of the burden of PTSD symptoms in patients with psychotic disorders experiencing coercive interventions in acute settings and shall be recommended as a measure of trauma-informed care.

The trial was registered at ClinicalTrials.gov (ID NCT03512925) on 01/30/2018 (retrospectively registered).

## Introduction

Coercion in psychiatric care has been increasingly the focus of clinical and scientific attention, mainly due to legal, ethical and clinical issues raised by the use of coercive measures such as mechanical restraint or seclusion. Although their life-saving potential is undisputed in emergency scenarios such as a delirium tremens, their use should be restricted to situations in which other alternatives have been exhausted [1]. Moreover, the known potential consequences of coercion on clinical outcomes, therapeutic relationship or satisfaction with care render the need to reduce their application urgently [2].

Concurrently, the presence and management of trauma experiences and related post-traumatic stress disorder (PTSD) among patients suffering from severe mental disorders such as psychosis has raised much attention over the last decades. Previous works showed very high rates of traumatic experiences such as sexual abuse and experience of violence among patients suffering from psychoses [3, 4]. In addition, there is a growing number of research works focusing on the relationship between trauma, psychosis and PTSD, with some authors suggesting that psychosis could be a way of reacting to traumatic experiences [5–7]. Moreover, the role of experiences made in psychiatric inpatient care, including involuntary admissions and coercive measures such as restraint or seclusion, has been examined, and studies suggest a potentially negative influence of coercive measures and other experiences in inpatient setting on the development or exacerbation of PTSD symptoms or underline traumatic experiences as a potential risk factor for experiencing coercive measures [8–11]. Findings suggest that a particular group of patients suffering from severe mental illness and having experienced traumatic events in the past could be particularly vulnerable to interventions that might precipitate or exacerbate symptoms of PTSD. Paksarian et al. also showed that women were more likely to report having experienced traumatic events during past hospital stays, a finding in line with other works showing that women were more likely to report harmful experiences in psychiatric settings and negative impact of coercion [10, 12]. Hence, interventions are needed that not only aim at reducing the use of coercion but also address trauma-related issues.

Among strategies implemented to reduce the use of coercive measures in inpatient care, post-coercion review sessions have received growing attention. Through a joint analysis and reflection of the situation that led to the coercive measure, goals of post-coercion review are: to allow patients and staff members to view the event from the others’ perspective, to repair ruptures of the therapeutic alliance and to reinforce working relationships, provide emotional expression and relief regarding the experienced situation and coercive measure, and to prevent the use of further coercive interventions^.^[13, 14]. Post-coercion review sessions have been evaluated as an important intervention in the context of coercion by patients and professionals [15]. Precise guidelines or recommendations on the content or performance of post-coercion reviews have not been published, and they are rarely performed in clinical practice according to Needham and Sands [16]. To date, one other controlled study investigating the effects of post-coercion review did not show a significant reduction of PTSD symptoms [17]. Based on the previous clinical experiences made within the context of a recovery orientation of inpatient care (Weddinger Model), a standardized guideline for the conduction of a post-coercion reflecting review session was developed [13, 18]. A first observational study indicated a good acceptance of the intervention by patients and staff members [13, 14].

### Aims of the study

The main goal of this work was to determine the impact of standardized post-coercion review sessions on the experience of peritraumatic distress and posttraumatic symptoms in patients with psychotic disorders who underwent coercive measures in an inpatient setting using a randomized controlled trial design. It was hypothesized that the provision of a single, standardized, post-coercion review session, compared to standard care without such a structured intervention, would reduce peritraumatic distress as well as the prevalence of PTSD symptoms at the time of discharge from hospital.

## Materials and methods

### Design

The present study is part of a larger RCT primarily conducted to investigate the effects of post-coercion review sessions on coercion-related outcomes (ClinicalTrials.gov-ID NCT03512925) financed by the German Ministry of Health. This sub-study examined the effect of a standardized post-coercion reflecting review session on the perception of coercive measures as potentially traumatizing and on the prevalence of PTSD symptoms at discharge following experienced coercion. The authors assert that all procedures contributing to this work comply with the ethical standards of the relevant national and institutional committees on human experimentation and with the Helsinki Declaration of 1975, as revised in 2008. All procedures involving patients were approved by the ethics committee of the Charité Universitätsmedizin Berlin (ID: EA1/158/17). The data that support the findings of this study are available from the corresponding author upon reasonable request.

### Participants

Patients admitted to acute psychiatric wards in 6 psychiatric clinics in Berlin were recruited between November 2017 and May 2019. We included patients with diagnoses of psychotic disorders (ICD-10 codes: F1×.5, F2×, F30.2, F31.2), aged between 18 and 65 years, who had experienced at least one coercive intervention during their current hospitalization. Participants had to be able to consent to their participation at the time of the assessment interview. Patients who were discharged within 24 h after admission were not included. Comorbid severe organic brain disorders, severe cognitive deficits and insufficient German language skills were exclusion criteria.Written informed consent was obtained from all participants.

### Definition of coercive interventions

The following coercive interventions were considered in the study: mechanical restraint, seclusion and forced medication based on court order.

### Participating clinics

All public psychiatric hospitals in Berlin were contacted to achieve study participation through their heads of department. Six centers which, respectively, provide psychiatric care for a defined catchment area responded positively. As all centers work under the same legislation and in the same county, homogeneity of standards and policies can broadly be assumed. The patient recruitment took place on the acute wards of the participating centers, where the vast majority of patient with severe mental illness are treated and coercive measures are predominantly executed.

Contact persons responsible for the recruitment were appointed on each participating ward. These contact persons ensured the planning and performance of the intervention.

### Recruitment, randomization and course of study

The contact persons on each ward were reached via telephone on every weekday to identify patients meeting the inclusion criteria. Data regarding age, sex, the type of coercive measure experienced, and the diagnoses were extracted. Since the planned intervention did not decisively differ from the usual routine of care and most potential study participants were unable to consent to study participation at the time of the first coercive intervention, we decided to conduct a randomization procedure suggested by Zelen to avoid recruitment bias [19, 20]. We used block randomization with periods of 8 for each ward. Randomization blocks were generated by the main research investigator using an online randomization tool. Patients were allocated to either the intervention or the control group immediately after they experienced the first coercive intervention during their hospital stay. The randomization result was communicated to the contact person on each ward and, thus, unmasked to staff members, research workers and patients. As the assessment interviews were centered on the effects of the post-coercion review session on the experience of coercion, research personnel were unblinded with regard to the randomization status. The sample size regarding the primary outcomes of perceived coercion studied in our RCT was calculated on the basis of an expected medium effect size (Cohen’s *f* 0.25), an expected power = 0.80 and planned factorial ANCOVA with two factors and a covariate. A sample size of *n* = 128 was calculated.

The daily telephone contacts also served as an opportunity to establish whether the reflecting review sessions (intervention group) were conducted and to organize the assessment interview that took place at discharge from the ward and was completed by trained research assistants of the main research team. Therefore, they were not involved in patients’ treatments. At that time, patients were asked to give their written informed consent to participate.

The contact persons on the wards informed the research team about execution and date of the reflecting review sessions. Participants were asked during the assessment if they had received the scheduled intervention and were invited to give brief descriptions. Patients who had been randomized to the control condition were equally questioned whether they had a post-coercion conversation with a team member.

### Description of study intervention: reflecting review session

Participants randomized to the intervention group were offered the opportunity to participate in a standardized, post-coercion reflecting review session during their hospital stay. This interview was conducted by staff members who underwent a previous training course. Intervention guideline, frame and setting described by Wullschleger et al. served as the basis of this structured intervention [14]. Besides the patient, a staff member actively involved in the decision to use coercion participates to the session and patients are encouraged to invite any person of trust or another member of staff or peer-worker to participate. The session is moderated by a member of staff not directly involved in the coercive situation. The moderator conducts the interview, hereby guaranteeing the structure and completion of the interview, as well as inviting the patient to express his or her perception and feelings about the coercive measure and the precipitating situation. Participants are first asked to describe their perception of the escalating crisis situation which lead to the eventual use of coercion and the coercive measure itself. Therefore, a process of sharing of patients’ and staff members’ perspectives is initiated. Then, the moderator asks open-ended questions addressing following issues: alternatives to coercion, personal wishes during and after the coercive intervention, intelligibility of the reasons for the use of coercion. At the end of the interview, the patient is offered the opportunity to include the conclusions of the interview in a joint crisis plan or an advance directive.

The interview was repeatedly offered to the patient until his/her discharge from the ward, as the pilot study had shown that patients themselves should determine the preferred point of time to discuss their experience of coercion.

Although initially designed as a “debriefing intervention” supposed to be performed promptly after the initial coercive intervention took place, the pilot evaluation had also pointed out that most patients were emotionally and clinically incapable to join this interview until a later point of their hospital treatment. For this reason, we decided to rename the intervention into “post-coercion reflecting review session” to underline its reflecting character and avoid confusion with other debriefing interventions.

#### Training and implementation

A training course providing the adequate application of the guideline was developed. It consisted of the presentation of the theoretical and scientific background of the intervention, the description of the guideline and a role play. This course was given to the treating multi-professional teams in the participating centers prior to the initiation of the study.

### Description of the control intervention: standard treatment

Patients who were randomized to the control group underwent routine clinical treatment which might have encompassed conversations about experienced coercion and the therapeutic processing of their personal and emotional sequelae. However, these interventions were administered in an unstructured manner based on clinical necessities lacking a standardized frame and setting.

### Measures

#### Socio-demographic and anamnestic data

Information on age, sex, socio-economic status and history of migration were collected during the assessment interview. Clinical data regarding previous hospitalizations, present and past pharmacological treatment, substance abuse, former experiences of coercion and debriefing interventions were captured.

#### Clinical data

The treating clinicians were asked to complete the Global Assessment of Functioning scale (GAF) and the Clinical Global Impression Severity scale (CGI-S) for each participant regarding their mental state at the time of the first coercive intervention [21, 22]. To simplify the assessment of symptoms and reduce the proportion of missing data, clinicians rated the severity of the following symptom categories on individual 4-point Likert scales (absent, mild, moderate, severe): positive symptoms, negative symptoms, global symptomatology, mania, depression and lack of insight.

#### Objective use of coercion

Data on type and number of coercive interventions experienced by the study participants during the index hospitalization were collected by reviewing patients’ records.

#### Perception of coercive measures as distressing and potentially traumatizing

We used the German version of the Peritraumatic Distress Inventory (PDI), which was developed to assess the PTSD diagnostic criterion A2 of the DSM-IV TR [23]. The PDI measures the level of emotional distress and physiological reactions experienced during or immediately after a traumatic event. It comprises 13 items rated on 5-point likert scales and a total score is composed by adding the scores obtained for each item. Higher values indicate a higher intensity of the peritraumatic reaction and thus a higher risk to develop PTSD. A cut-off score of 14 has been proposed to identify patients at risk, thus needing further assessment (sensitivity 84% and specificity 47%) [24].

#### PTSD symptoms

Symptoms of PTSD were assessed with the help of the German version of the Impact of Events Scale-Revised (IES-R) [25]. The IES-R is an instrument designed to assess the presence of symptoms of PTSD. Patients rate each of the 22 items on a 4-point Likert scale according to the frequency of presented symptoms. Three subscales are formed: (1) intrusion, (2) avoidance and (3) hyperarousal. Higher values on each subscale indicate a higher level of symptom load. According to Maercker et al., the presence of PTSD can be assessed as follows using the three IES-R subscale scores: *X* = (− 0.02 × Intrusion) + (0.07 × avoidance) + (0.15 × hyperarousal)  − 4.36. If *X* > 0, a PTSD should be suspected [26]

### Statistics

Socio-demographic and clinical characteristics of the studied samples were compared using *t *test and chi-square tests.

An intention-to-treat analysis was performed based on the randomization results, regardless of violations of the study protocol. In an exploratory analysis, we investigated the possible influence of the kind of experienced coercive measure on the results, as some evidence indicates that seclusion might be better accepted than restraint, although data regarding this aspect refer to indicators of subjective perceived coercion or patients’ preferences and no evidence clearly points at differences between seclusion and restraint as to potential adverse effects including the development of PTSD [27]. No significant differences were found regarding the tested outcomes and so the variable was not included in the main analysis. Similarly, differences between participating clinics were investigated without significant results.

A univariate ANOVA was performed to assess the main effects of the independent factors post-coercion reflecting review session and gender as well as their interaction effect on the peritraumatic reaction elicited by the index coercive measure. A factorial MANCOVA and post hoc univariate ANCOVAs were used to investigate the main effects of the independent factors post-coercion reflecting review session and gender as well as their interaction effect on post-traumatic symptoms as measured by the 3 subscales of the IES-R as dependent variables. To control for the effect of the perception of the coercive measure as traumatic the mean PDI score was used as a covariate. A Chi-square test was conducted to compare the risk of developing a PTSD based on the proposed cut-off score for the PDI [24]. A Chi-square test was also executed to assess differences of the clinical probability of having a PTSD based on the IES score proposed by a formula of the German translators [26].

Statistical significance was defined at a two-sided *p* < 0.05. IBM SPSS Statistics 25 was utilized for statistical calculations.

## Results

### Sample description

A total number of 422 patients were randomized after having experienced a coercive intervention on one of the participating wards. 211 patients were initially allocated to the intervention and 211 to the control group. In each group, 98 patients were discharged unexpectedly before being contacted by the research team. Among those contacted, 35 patients in the intervention group and 40 in the control arm refused participation. Respectively, 26 and 16 patients in the intervention and control group were excluded because of persisting cognitive deficits, language barrier or adjustment of their main diagnosis. Finally, 109 patients consented to participate—52 participants in the intervention group and 57 in the control group. 100 patients (intervention group *n* = 45; control group *n* = 55) answered the PDI and 83 the IES-R (intervention group *n* = 36; control group *n* = 47). A total of 82 participants answered both the PDI and the IES-R (intervention group *n* = 36; control group *n* = 46) and, thus, constituted the final sample for intention-to-treat analysis.

**Fig. 1:**
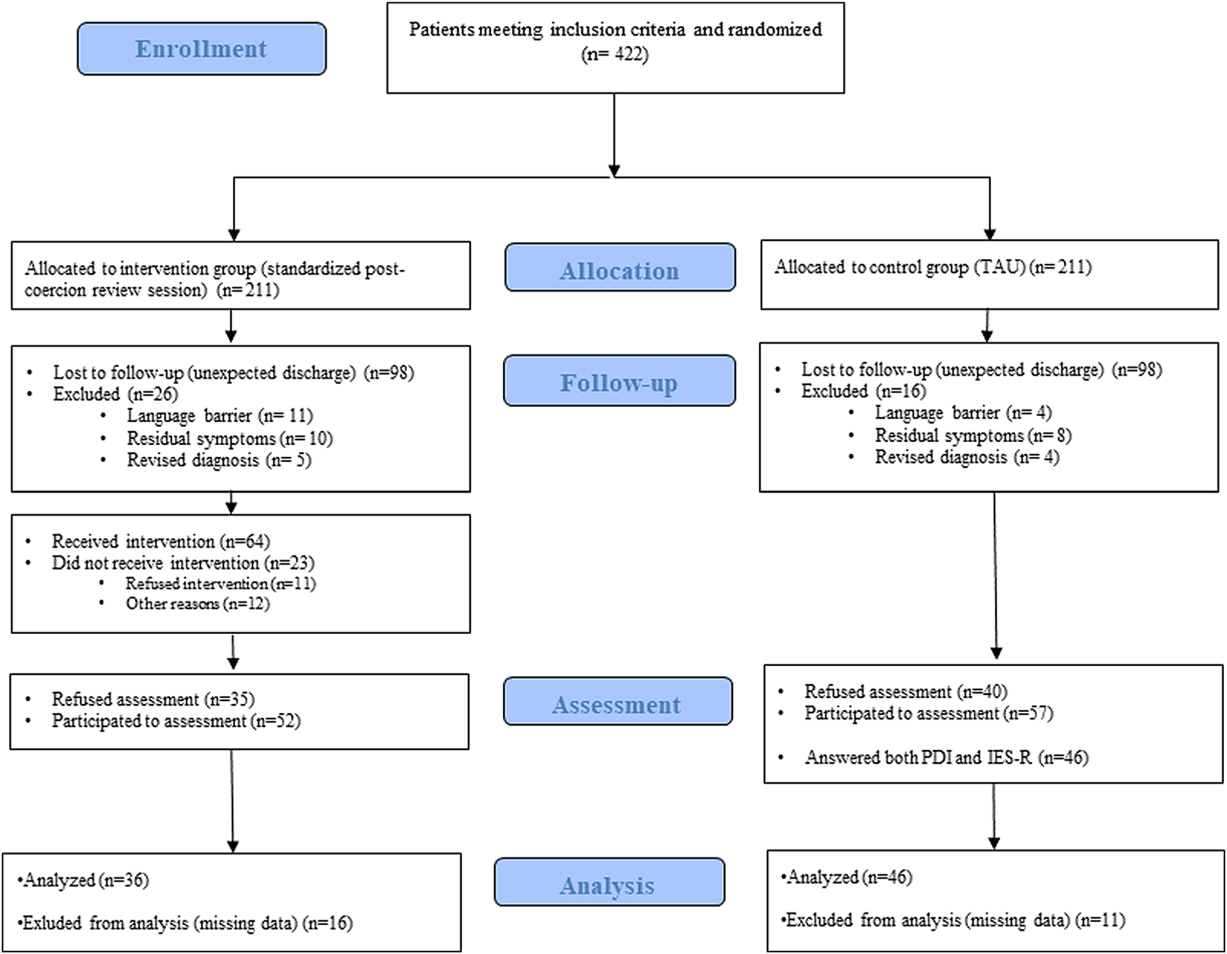
Study flowchart (adapted from the CONSORT diagram)

Among them, 32 participants in the intervention group received a post-coercion reflecting review session as planned. In the control group, 24 patients reported not receiving any kind of post-coercion review. The randomization chart is shown in Fig. 1.

The socio-demographic and clinical characteristics of the included participants are summarized in Table 1. No statistical differences were found between the samples regarding socio-demographic or clinical data.

**Table 1:** Socio-demographic and clinical characteristics of the studied samples

	Control *n* = 46	Intervention *n* = 36	Total *n* = 82
Age (years) M (SD)	38.89 (10.98)	39.14 (14.87)	39.00 (12.75)
Gender *n* (%)			
Female	21 (45.7%)	22 (61.1%)	43 (52.4%)
Male	25 (54.3%)	14 (38.9%)	39 (47.6%)
Hist. of migration *n* (%)	*n* = 45	*n* = 35	*n* = 80
Yes	7 (15.6%)	12 (34.3%)	19 (23.7%)
No	38 (84.4%)	23 (65.7%)	61 (76.3%)
Incap. benefits *n* (%)	*n* = 45	*n* = 33	*n* = 78
Yes	15 (33.3%)	10 (30.3%)	25 (32.1%)
No	30 (66.7%)	23 (69.7%)	53 (67.9%)
Level of education *n* (%)	*n* = 45	*n* = 31	*n* = 76
No degree	3 (6.7%)	1 (3.2%)	4 (5.3%)
Lower sec. education	7 (15.6%)	4 (12.9%)	11 (14.5%)
Higher sec. education	13 (28.9%)	9 (29.0%)	22 (28.9%)
High school graduation	8 (17.8%)	5 (16.1%)	13 (17.1%)
Vocational college	7 (15.6%)	6 (19.4%)	13 (17.1%)
University	7 (15.6%)	6 (19.4%)	13 (17.1%)
Diagnosis *n* (%)			
F19.×5, F30.2, F31.2	8 (17.4%)	10 (27.8%)	18 (22.0%)
F2.×	38 (82.6%)	26 (72.2%)	64 (78.0%)
Clinical parameters	*n* = 39	*n* = 33	*n* = 72
GAF M (SD)	29.15 (12.40)	26.58 (14.54)	27.97 (13.39)
CGI-S M (SD)	5.59 (.72)	5.73 (.63)	5.65 (.67)
Symptom severity M (SD)			
Positive sympt.	2.41 (.79)	2.12 (1.02)	2.28 (.91)
Negative sympt.	1.26 (.91)	1.18 (0.85)	1.22 (.88)
Global sympt.	2.41 (.68)	2.36 (.70)	2.39 (.68)
Mania	1.36 (1.11)	1.24 (1.30)	1.31 (1.19)
Depression	.54 (.85)	0.42 (.66)	0.49 (.77)
Lack of insight	2.41 (.82)	2.27 (.91)	2.35 (.86)
Past coercion *n* (%)	*n* = 45	*n* = 36	*n* = 71
Yes	31 (68.9%)	26 (72.2%)	57 (70.4%)
No	14 (31.1%)	10 (27.8%)	24 (29.6%)
Previous post-coercion review *n* (%)	*n* = 31	*n* = 27	*n* = 58
Yes	3 (9.7%)	4 (14.8%)	7 (12.1%)
No	28 (90.3%)	23 (85.2%)	51 (87.9%)
Index coercive intervention *n* (%)			
Restraint	29 (63.0%)	23 (63.9%)	52 (63.4%)
Seclusion	12 (26.1%)	12 (33.3%)	24 (29.3%)
Forced med. on court order	5 (10.9%)	1 (2.8%)	6 (7.3%)
Coercive interventions during stay			
Restraint			
Patients *n* (%)	32 (69.6%)	24 (66.7%)	56 (68.3%)
Events M (SD)	1.53 (.95)	2.28 (3.21)	1.90 (2.24)
Seclusion			
Patients *n* (%)	31 (67.4%)	25 (69.4%)	56 (68.3%)
Events M (SD)	1.81 (1.42)	2.40 (3.12)	2.07 (2.33)
Forced med. on court order			
Patients (%)	3 (6.5%)	4 (11.1%)	7 (8.5%)

*M *mean*, SD* standard deviation,* GAF* global assessment of functioning, *CGI-S *clinical global impression-severity scale

On average, the post-coercion reflecting review session took place at a median of 34.5 days after the initial coercive measure.

Patients who refused to participate were slightly older (44.23 vs. 38.83 years.) than participating patients and the female proportion was marginally larger (52% vs. 49.54%). Regarding the patients in the intervention arm who refused to participate, 16 received a post-coercion reflecting review session, 9 refused it and 10 patients did not receive the intervention for other reasons (time limitation, intervention not provided by the team).

### Peritraumatic reaction

Mean PDI values are summarized in Table 2.

**Table 2:** Mean values of the PDI and the IES-R subscales across the study groups

	Control (*n* = 46)	Intervention (*n* = 36)	Total (*n* = 82)
PDI			
Mean	23.65	22.03	22.94
SD	15.36	11.67	13.81
IES-R			
Intrusion			
Mean	13.48	7.97	11.06
SD	11.42	8.55	10.56
Hyperarousal			
Mean	13.11	8.92	11.27
SD	10.20	7.55	9.32
Avoidance			
Mean	17.35	17.50	17.41
SD	12.78	11.39	12.11

*PDI * Peritraumatic Distress Inventory, *IES-R *Impact of Events Scale-Revised, *SD* standard deviation

The mean PDI score of patients in the intervention group was 22.03 (SD = 11.67) and 23.65 (SD = 15.36) in the control group. The performed ANOVA showed no significant main effect of the intervention or gender and no significant interaction effect of intervention and gender. Results are shown in Tables 2 and 3.

**Table 3:** Univariate ANOVA and ANCOVA results for the PDI and the IES-R subscales

	SS	*df*	MS	*F*	*P*	Part*. η*^2^
PDI						
Intervention	115.67	1	115.67	0.60	0.440	0.008
Gender	323.72	1	323.72	1.69	0.198	0.021
Intervention × gender	136.28	1	136.28	0.71	0.402	0.009
Error	14,981.16	78	192.07			
Total	58,591.00	82				
IES-R intrusion						
PDI	3589.56	1	3589.56	57.24	< 0.001*	0.426
Intervention	360.12	1	360.12	5.74	0.019*	0.069
Gender	66.95	1	66.95	1.07	0.305	0.014
Intervention × gender	55.53	1	55.53	0.89	0.350	0.011
Error	4829.12	77	62.72			
Total	19,067.00	82				
IES-R hyperarousal						
PDI	2835.87	1	2835.87	57.30	< 0.001*	0.427
Intervention	215.64	1	215.64	4.36	0.040*	0.054
Gender	2.69	1	2.69	0.05	0.816	0.001
Intervention × gender	37.65	1	37.65	0.761	0.386	0.010
Error	3810.12	77	49.48			
Total	17,454.00	82				
IES-R avoidance						
PDI	3313.16	1	3313.16	30.73	< 0.001*	0.285
Intervention	13.49	1	13.49	0.13	0.724	0.002
Gender	42.58	1	42.58	0.40	0.532	0.005
Intervention × gender	93.07	1	93.07	0.863	0.356	0.011
Error	8302.34	77	107.82			
Total	36,752.00	82				

Mean PDI score used as covariate

*IES-R* Impact of Events Scale-Revised, *PDI* Peritraumatic Distress Inventory, *SS* sum of squares, *df* degrees of freedom, *MS *mean square, *F *ANCOVA *F *statistic

*p < 0.05

Using the cut-off score of 14, the analysis showed that 30 patients in the control group (65.2%) and 27 patients (75.0%) in the intervention group exhibited a peritraumatic reaction requiring further clinical assessment regarding the risk of developing a PTSD. Difference across groups was not statistically significant, *X*^2^(1) = 0.912, *p* = 0.340.

### Symptoms of PTSD

Mean values of all three IES-R subscales are summarized in Table 2.

The performed multivariate analysis (MANCOVA) across all three IES-R subscales with intervention and gender as independent factors and the mean PDI score as covariate showed a significant effect of the intervention at the multivariate level, Pillai’s trace = 0.109, *F*(3,75) = 3.054, *p* = 0.034, partial *η*^2^ = 0.109. The covariate (mean PDI score) proved to be significantly correlated with the analyzed dependent variables at the multivariate level, Pillai’s trace = 0.489, *F*(3,75) = 23.901, *p* < 0.001, partial *η*^2^ = 0.489. Neither gender nor the interaction between intervention and gender showed statistically significant effects at the multivariate level [gender: Pillai’s trace = 0.034, *F*(3,75) = 0.887, *p* = 0.452; intervention × gender: Pillai’s trace = 0.016, *F*(3,75) = 0.415, *p* = 0.743].

Subsequent univariate ANCOVAs using the different IES-R subscales as dependent variables, intervention and gender as independent variables and mean PDI score as a covariate were performed. Results are summarized in Table 3. There was a statistically significant main effect of the intervention for the subscales intrusion and hyperarousal, with participants in the intervention group showing lower mean scores on these subscales. No main effect of the intervention was found regarding the avoidance subscale. Furthermore, no main effect of gender or of the interaction between the two independent variables was found. The effect of the covariate was shown to be statistically significant across all three subscales, with higher mean PDI scores being associated with higher scores on the IES-R subscales.

### Clinical probability of PTSD

When analyzing the clinical probability of PTSD across the studied sample using the formula proposed by Maercker et al., results highlight that 13 patients (28.3%) in the control group and 4 (11.1%) in the intervention group showed a high diagnostic probability of having a PTSD [26]. This difference, however, was not statistically significant, *X*^2^ (1) = 3.614, *p* = 0.057.

## Discussion

The results of this RCT suggest for the first time a beneficial effect of post-coercion review sessions on the development of certain symptoms that might be indicators of the development of PTSD after coercive interventions in patients with psychotic disorders. The performed analysis indicated that patients who underwent a standardized post-coercion review showed significantly lower levels of intrusion and hyperarousal symptoms as measured by the IES-R. Accordingly, a lower proportion of probable PTSD was found among patients who received the intervention compared to the control group. This difference was, however, only marginally significant, which is most probably linked to the fact that avoidance symptoms were not affected by the intervention and to a lack of statistical power. These findings, thus, highlight that post-coercion reviews might be a means of counteracting the negative effect of coercive measures on these symptoms known to be invalidating and pervasive in some patients.

This result is not in keeping with the single previous study on this issue [17]. However, the study of Whitecross et al. examined patients with psychoses as well as other psychiatric disorders regarding their experience of seclusion. The study design was controlled, but not randomized, and intervention and control conditions were implemented on different wards. Even though a similarly high proportion of patients met the criteria for ‘probable PTSD’ on the IES-R, post-seclusion counseling did not reduce the trauma experiences significantly compared to control patients who were not offered this intervention. Differences to our findings might not only be explained by a larger sample size and a more rigorous design in the present study, but also by examination of different coercive measures (seclusion, restraint, forced medication). It can be assumed that mechanical restraint and forced medication bear a higher traumatic impact compared to seclusion, which may render a respective intervention more effective. Moreover, the post-coercion review in our trial was delivered much later in the course of the inpatient treatment (43 days after the initial coercive measure versus 3–7 days post-seclusion in Whitecross et al.). The nurses’ interventions of Whitecross et al. were based on five essential areas of debriefing (counseling; ventilation; support and reassurance; screening for physical adverse effects; psychoeducation), while setting and content of the multi-professional review session reported here are considerably different, putting the focus on mutual perspective taking and repair of a ruptured working relationship with the team in presence of a moderator ensuring proper conduction of the interview.

Therefore, it can be discussed that the effect of the present intervention relates to its particular setting and its psychotherapeutic character. The close involvement of patients and the encouraged dialog with the staff members facilitates differentiation of emotions and exchange of subjective perceptions of the coercive situation. The given opportunity to repair the potentially damaged therapeutic relationship and to restore trust and respect being essential for self-worth and -efficacy (post coercion review denotes the option of joint crisis plans) in spite of the coercive intervention might be additional factors contributing to the reduction of PTSD symptoms.

Beyond these results, this study confirmed the highly traumatic potential of coercive measures. Overall, about 70% of the included patients presented distinct peritraumatic reactions rendering them at risk of developing a PTSD. Accordingly, about 20% of the participants showed a high clinical probability of PTSD. These results are in line with previous works investigating the deleterious effect of coercive measures and traumatic experiences made within psychiatric settings [8, 9]. They, thus, underline the necessity of a thorough assessment of trauma-related symptoms, particularly in conjunction with coercion. The negative and potentially traumatic experiences made during inpatient therapy might have serious consequences on clinical course, engagement into treatment and recovery perspectives. Moreover, the high prevalence of traumatic experiences during hospital treatment is not compatible with a human rights’ perspective in psychiatric care [28]. Reducing coercive interventions in psychiatry must, therefore, be considered an ethical and clinical imperative.

### Limitations

A number of limitations might have influenced our results. The study design did not encompass the assessment of previous traumatic experiences that might have been made outside of the psychiatric context, during previous inpatient hospitalizations or through the experience of psychotic states. The possible association between these previous experiences and the severity of the reaction to coercive interventions should be studied in further works. The retrospective assessment of the peritraumatic reaction and PTSD symptoms weeks after the coercive intervention took place could also be considered as potential bias, as events that followed the coercive measure and that took place during the hospital stay might have influenced responses. However, the findings of the present study regarding the prevalence of PTSD are in line with previous works and it can, thus, be assumed that this bias did not significantly affect the results [29].

Another limitation refers to the inclusion rate of patients which did not allow the research team to meet the expected inclusion goals during the planed recruitment period and, thus, resulted in a loss of power. Unfortunately, a relevant number of patients were not reached by the research team prior to their prompt or unplanned discharges. Willingness to participate in this study may have been associated with younger age as a potential indicator of lower chronicity. As selection bias is not fully avoidable in this and similar investigations, the studied sample must not be considered entirely representative of the inpatient population experiencing coercive measures. Future evaluations of post-coercion review should ensure that briefly hospitalized patients are receiving the foreseen intervention. A stronger focus on staff training or a stable team of moderating staff members might be useful to achieve this goal. The assessment of long-term effects of post-coercion review on PTSD symptoms and the development of manifest PTSD itself shall be focused on in future research.

In summary, the developed standardized post-coercion review can be seen as an intervention that might contribute to the reduction of the burden of PTSD symptoms in severely ill patients subjected to coercive interventions. It can be implemented without greater effort and serves as an important tool to strengthen trauma-informed care in inpatient settings.

## Data Availability

Data are available through the corresponding author upon reasonable request.
